# Indocyanine-green-assisted near-infrared dental imaging - the feasibility of *in vivo* imaging and the optimization of imaging conditions

**DOI:** 10.1038/s41598-019-44660-y

**Published:** 2019-06-03

**Authors:** Zhongqiang Li, Shaomian Yao, Jian Xu

**Affiliations:** 1 Division of Electrical and Computer Engineering, College of Engineering, Louisiana State University, Baton Rouge, LA70803 USA; 2 Department of Comparative Biomedical Science, School of Veterinary Medicine, Louisiana State University, Baton Rouge, LA70803 USA

**Keywords:** Endoscopy, Fluorescence imaging, Optical imaging, Dental diseases

## Abstract

X-ray-based imaging, including computed tomography, plays a crucial role in the diagnosis and surgery of impacted teeth that affects over 25% of the human population. But the greatest disadvantage of this technique is ionizing radiation risk to the patients. Here we describe a completely ionizing-radiation-free *in vivo* near-infrared (NIR) fluoresence dental imaging with indocyanine green (ICG) agent that has rarely been applied in dental imaging. Our method can acquire dental structure images within a short period (only 10 minutes after injection) without ionizing radiation risk. NIR enables the observation of dental structures that are not distinguishable under visible conditions. At prolonged 72 hours, only molar regions remained highlighted; the contrast between molar regions and surrounding tissues was prominent; this is particularly useful for *in vivo* dental imaging. Using the quantitative spectral analysis, we found the peak wavelengths of ICG fluorescence shifted along with the injection time: the peak wavelength shifted 8 nm (from 819 nm to 811 nm) in 0~72 hours. The injection methods of tail vein *v.s*. intradermal injections caused ~3 nm shift. ICG-assisted NIR fluorescence imaging can serve as a useful tool for *in vivo* real-time diagnosis in dental clinics and surgeries without ionizing radiation risk.

## Introduction

It was estimated that over 25% of the human population suffered from impacted teeth (delayed or failed eruption); the highest incidence occurs on the third molar^1,2^. X-ray imaging is the most common diagnostic tool for clinical examination of patients with impacted teeth^3–5^. Particularly, computed tomography (CT) is used in three-dimensional (3D) visualization of tooth structures^3–5^. To date, there is a great need for patients to take routinely dental imaging. Dental radiography contributes almost one-third of total radiological examinations in western countries^6^; for example, American Dental Association (ADA) recommends that dental imaging should be taken at least once every 1~3 years^7^. However, the greatest disadvantage of X-ray imaging is the ionizing radiation exposure causing ionizing effects on human tissues, which may lead to killing or malfunctioning of cells at high doses^8^. X-ray radiation exposure may also be attributable to ~2% of invasive cancer incidents^9,10^. In dentistry, X-ray radiation risks are of higher concern to children, who on average have to take X-ray radiation 3~6 times more frequently than adults, due to their rapid rates of teeth growth and decay^7,8,10^.

There are a few non-ionizing-radiation dental imaging methods in development, Magnetic Resonance Imaging (MRI) is considered to be safe for 3D dental imaging without ionizing radiation risk^3^. However, due to its high cost, its use in dentistry is limited to assess precise diagnostics for exceptional cases^3,11^. Ultrasound (US) imaging is another non-invasive, inexpensive and painless method^3^; however, this method has limitations in detecting the periodontal ligament and in diagnosing fractures^3,12^. Additionally, optical coherence tomography (OCT) becomes popular in dental research because of its safety, noninvasive imaging, excellent spatial resolution (~20 μm), etc^13^. Nonetheless, it is limited to a restricted scanning range due to low penetration depth^13^. Therefore, it will be of great significance to develop an efficient and easy-to-use dental imaging technique for intraoperative diagnosis in dental surgeries without ionizing radiation risk.

Near-infrared (NIR) imaging, especially fluorescence imaging, plays an essential role in many areas of biomedical sciences^14,15^. In dentistry, existing research focuses on using NIR light, with a wavelength of 1310 nm, to acquire high imaging contrast between the caries lesions and sound teeth by illumination^16,17^. Few studies reported using NIR light with the enhancement of indocyanine green (ICG) to image dental tissues of impacted teeth^18^. ICG, approved by Food and Drug Administration (FDA) for clinical uses^19^, is known to produce NIR fluorescence (650–950 nm) in angiography^14,20^. Currently, ICG is widely used in retinal angiography, cancer surgical imaging, lymph-node detection, etc^19,21,22^. ICG was reported to serve as a photosensitizer dye or a photo-absorbing dye for dental treatments^23,24^.

Meanwhile, endoscopic imaging under NIR condition could provide significant information regarding tumors (e.g. feeding artery) than that of wide-field imaging^19^. When used in dentistry, the endoscopy not only helps to significantly reduce the patient pain, but also helps them to recover from dental surgery^25^. To our best knowledge, ICG-enhanced endoscopic dental imaging has not been systematically investigated^18,26^. Although in a pilot study we demonstrated that dental structures of animal models can be imaged *ex vivo* with a lab-designed ICG-enhanced endoscopic dental imaging system^18,26^, this work represents the first effort to study the feasibility of this approach to generate *in vivo* NIR dental images of the developing molars of postnatal rats and to explore the potential factors that can be optimized to improve the imaging quality.

## Results

### *In vivo* NIR dental imaging of unerupted molars

**Figure 1:**
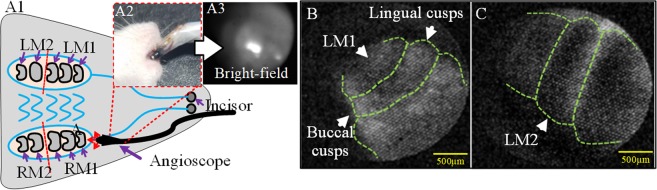
*In vivo* NIR dental imaging of unerupted molars: (**A1–A3**) the schematic diagram and bright-field photographs of the unerupted molar observed by angioscope; (**B**,**C**) the NIR fluorescence images of the P14 molars, taken by camera with the angioscope. LM1: left first molar; LM2: left second molar; RM1: right first molar; RM2: right second molar. Green lines: molar sketch.

In the *in vivo* dental imaging, one P14(postnatal 14 days) rat was imaged after 72 hrs of ICG intradermal injection via the intradermal method. The deflecting tip (front end) of the angioscope was inserted into the rat oral cavity and was moved forward and backward to detect the molars *in situ* (Fig. 1A1–A3). Under visible conditions (bright-field), only a bright reflection spot was seen and no molar structure profiles were obtained from the bright-field imaging identified (Fig. 1A2).

With our ICG-assisted NIR imaging system, through the angioscope, clear morphological profiles of the molars were observed *in situ* from the angioscopic fluorescent images, and even the cusps (raised points on the crowns of teeth) were unambiguously distinguished (Fig. 1B). By adjusting the imaging angle of the deflecting tip, more detailed morphology of the molars (such as buccal and lingual cusps) was identified from the fluorescence images (Fig. 1C).

### *Ex vivo* NIR dental imaging of unerupted molars

To obtain the *ex vivo* NIR dental images, one P14 rat was imaged at 10 min after injection. Two other P14 rats, including the P14 rat in the previous *in vivo* study, were imaged by our NIR imaging system after 72 hrs of injection. All three P14 molars were imaged under the wild-field and the sigmoidoscopic conditions.

**Figure 2:**
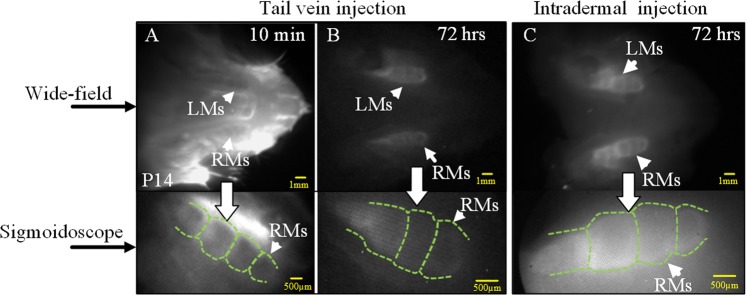
*Ex vivo* NIR dental imaging for the unerupted molars from P14 rats. (**A**) 10-min imaging window. (**B**,**C**) 72-hr imaging window. (**A,B**) tail vein injection of ICG. (**C**) Intradermal injection of ICG; the same P14 rat as in the *in vivo* imaging (Fig. 1). LMs: left molars; RMs: right molars. Green lines﻿: molar sketch.

In the wide-field imaging, dental structures of the P14 rat (Fig. 2A) at the 10-min imaging window (from the moment of ICG injection to the moment of observation) showed brighter fluorescence than that of the two P14 rats at the 72-hr imaging window (Fig. 2B,C).

The entire mandibular area was bright at 10-min after injection. In contrast, when the imaging window was prolonged to 72 hrs, only molar regions remained prominent for both injection methods (Fig. 2B,C), which facilitated the observation of dental structures.

In the endoscopic NIR imaging (Fig. 2B,C), the molar profiles of the P14 rats could be distinguished easily at 72 hrs after injection; the 10-min imaging window achieved even better imaging contrast; each cusp was recognized clearly from the endoscopic images (Fig. 2A).

### The impact of injection methods on NIR dental imaging

Two P9 rats were used to investigate the effect of different injection methods on NIR dental imaging. Specifically, one of the P9 rats was administered 10 µL (~0.5 mg/kg body-weight, ~0.3 mg/kg body-weight for P14 rats) ICG via the tail vein injection, while the other was injected by the intradermal injection. Both rats were imaged by NIR camera with the endoscope under visible and NIR conditions at 24 hrs.

**Figure 3:**
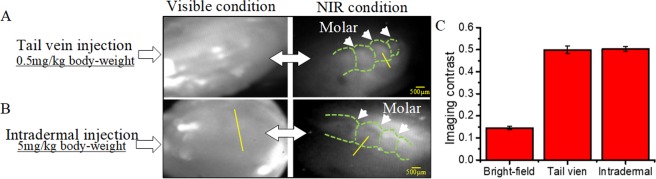
NIR dental imaging (bright-field imaging vs. NIR imaging) for P9 rats with (**A**) tail vein injection and (**B**) intradermal injection. (**C**) The grayscale difference imaging contrast between the molar regions and surrounding tissues. The pixels are sampled from the yellow lines. Green lines﻿: molar sketch.

Under the visible condition, the profiles of the molars were unable to be distinguished from the surrounding tissues (left images in Fig. 3A,B). However, under NIR condition, three cusps of the first molar were clearly recognized from the endoscopic fluorescence images (right images in Fig. 3A,B). From the quantitative analysis of grayscale difference, the bright-field imaging contrast was much lower than that of the NIR imaging, but the two injection methods did not show obvious difference on image quality (Fig. 3C).

### The effect of intraoral and extraoral excitations on NIR dental imaging

**Figure 4:**
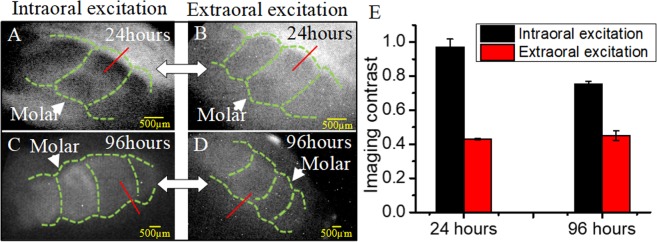
NIR dental imaging of the P14 rats with the intraoral and extraoral excitation; ICG was administered into the rats by the tail vein injection. The rats were sacrificed and imaged at 24 hrs (**A,B**) or 96 hrs (**C,D**) after injection. (**E**) Quantitative analysis on imaging contrast: the normalized grayscale difference between the molar regions and surrounding tissues, based on the data sampled from the red lines. Green lines﻿: molar sketch.

Two P14 rats with the tail vein injection were imaged at 24-hr and 96-hr imaging windows under either intraoral or extraoral light excitation conditions. When at the 24 hr imaging window (Fig. 4A,B), the profiles of P14 molars with the intraoral excitation showed better imaging contrast than that of the extraoral excitation.

When the imaging window was prolonged to 96 hrs, only the molar regions remained prominent (Fig. 4C,D) This facilitated the identification of molar structures, however, it should be noted that the image contrast slightly decreased at 96 hrs as compared to 24 hrs (Fig. 4C,D vs. Fig. 4A,B). For the grayscale difference, the intraoral excitation had a larger magnitude than that of the extraoral excitation for both imaging windows; the 24-hr imaging window had a larger difference than the 96-hrs imaging window (Fig. 4E).

**Figure 5:**
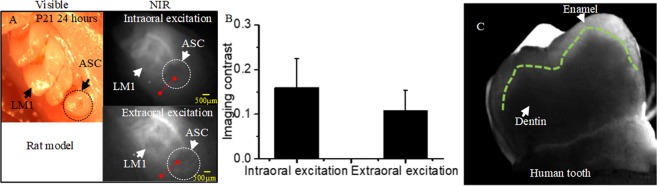
NIR dental imaging of the erupted 1st molars of a P21 rat, as well as extracted human tooth; the rat was administered by the intradermal injection and imaged at the 24-hr window; the extracted human tooth was immersed into ICG solution for 24 hours. (**A**) Bright-field images taken by microscopy, and the intraoral and extraoral excitation. (**B**) Quantitative analysis of imaging contrast between the molar regions and surrounding tissues, based on the data sampled from the red lines. (**C**) Sound human tooth under ICG-assisted NIR dental imaging. LM1: left first molar; ASC: Abnormally shaped cusp. Green lines: boundary of enamel and dentin.

In one of the P21 rats, we found an abnormally shaped cusp (ASC) in the left first molar (LM1) (Fig. 5A). To explore the impact of two ICG excitation methods on detecting abnormal molars, this P21 rat was imaged at the 24-hr window. Under NIR condition (Fig. 5A), the abnormal cusp could be clearly recognized by both the two excitation methods; the intraoral excitation demonstrated better imaging contrast to distinguish the abnormal cusp and the sound molar than that of the extraoral excitation. The grayscale difference also demonstrated quantitatively that the intraoral excitation had a better imaging contrast that did the extraoral excitation (Fig. 5B).

In addition, a sound extracted human tooth was immersed into 1 µM ICG solution for 24 hours to show the feasibility of ICG-assisted NIR dental imaging for human dentistry. From ICG-assisted dental image, the morphology of human tooth was able to be observed clearly; the enamel become transparent, while the dentin is relatively darker; the profiles of dentin were clearly delineated (Fig. 5C).

### Spectral analysis in NIR dental imaging

**Figure 6:**
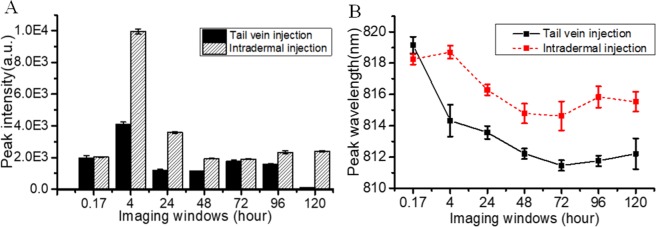
Characteristic of ICG spectra in NIR dental imaging. (**A**) The peak intensity of ICG spectra of P14 rats in the dental tissues (right) with the two injection methods changes at various imaging windows: 10 minutes (0.17 hr), 4 hrs, 24 hrs, 48 hrs, 72 hrs, 96 hrs, and 120 hrs. (**B**) The corresponding peak wavelength also shifts with the imaging windows.

Regarding the peak intensity, ICG fluorescence reached the maximum at 4 hrs after injection (Fig. 6A). ICG accumulation in the molar tissues by the intradermal injection was larger than that by the tail vein injection. After 24-hrs of injection, both injection methods had a significant decrease of ICG intensity within the imaging window. Thereafter, the magnitude kept relatively stable for both injection methods until 96 hrs after ICG injection. Finally, ICG intensity became nearly zero at 120-hr in the tail vein injection, while this value remained significant for the intradermal injection.

As for the peak wavelength shifts (Fig. 6B), both injection methods had the same peak wavelength (~819 nm) at the beginning, but there was ~3 nm difference after 24 hrs of injection. At 4 hrs after injection, the peak wavelength by the intradermal injection still remained ~819 nm, while the peak wavelength by the tail vein injection dropped to ~814 nm. After that, there was a slight blue-shift of ICG peak wavelength over time for both injection methods. Eventually, the peak wavelength stayed at 815 nm for the intradermal injection and 812 nm for the tail vein injection.

## Discussion

The first molars of rat are one of the most common animal models in studying odontogenesis, because of its similarity in limited eruption like humans^27^. Many findings and results obtained from rat molars have been applied successfully to human dental research and diagnosis^28^. Here, we reported that postnatal rats were injected with ICG agents; the NIR dental imaging system (camera + spectroscopic device), in conjunction with the endoscopes, was designed to observe, *in vivo* and *ex vivo*, the dental structures of postnatal rats after ICG administration. The results of this animal study showed that ionizing-radiation-free ICG-enhanced NIR dental imaging can be used to image dental structures of unerupted molars. This imaging technique has the potential for diagnosis of tooth eruption disorders and other dental abnormalities.

Imaging structures of unerupted molars is impossible by regular bright-field imaging since the molars are buried underneath the tissues^29^; observation of the morphology of an anomalously unerupted tooth is an integral part of diagnosis and treatment planning^29^. ICG-based NIR dental imaging can acquire clear tooth images for the unerupted molars in as short as 10 min after ICG injection (Fig. 2A). Within a 24-hr imaging window, the brightness of the surrounding tissues was relatively stronger than that of the unerupted molars (Fig. 2A).

At the prolonged imaging windows (72 hrs), only the molar area remained prominent in P14 rats (Fig. 2B,C). This could be due to the trapping of ICG agents in dental tissues because of the abrupt cessation in cell proliferation at later stages of tooth development^30^. A major advantage of this phenomenon is the *in vivo* dental imaging with the angioscope. This method can readily locate the molars and therefore reduce the time necessary for the procedure. The angioscopic *in situ* tooth images provide clear details on the morphology of the molars.

As seen in the *ex vivo* dental images of P9 rats, NIR improved the imaging quality significantly and helped to observe molar structures that were not recognized under visible conditions (Fig. 3). This indicates that our imaging system in the NIR range (800–950 nm) has a good tissue penetration depth likely due to the fact that NIR light in this range (650–950 nm) has lower absorption by blood, water, and lipids^31,32^. As a result, the signal-to-noise ratio in the imaging can be greatly enhanced, while the autofluorescence is minimized^31,32^. Therefore, ICG-based dental imaging is more convenient to use for imaging unerupted and impacted teeth, when compared to 1310 nm NIR light that is reported for dental imaging^16,17^.

Meanwhile, our results also demonstrated that both extraoral and intraoral ICG-excitation methods were able to observe unerupted molars and abnormally shaped cusp of erupted molars. The capability of clearly imaging dental tissues with intraoral illumination was a result of the good tissue penetration by the NIR range (800–950 nm) used in this study^31,32^. Additionally, the intraoral illumination is more likely to gather more fluorescence photons to generate clearer dental images. Although the current prevalent X-ray imaging and CT also have good tissue penetration, X-ray based dental imaging methods have several drawbacks, including radiation risks to the patients, complicated and expensive equipment and incapable real-time imaging^3–6^. This study suggests that ICG-enhanced NIR fluorescence dental imaging can be used as a safe (ionizing-radiation-free), portable and real-time imaging system for diagnosis and surgeries in dental clinics.

In human ICG-based imaging, low-dosage ICG agent is usually administered by the intravenous injection and is transported by the blood circulation^19,33^. For lymph-node imaging, ICG with the intradermal injection is transported via the lymphatic circulation^34^. In this study, we found that both the injection methods and the imaging windows were effective on features of ICG spectra (wavelength shifts). From the observation of peak intensity changing over time, the intravenous injection had faster excretion rate than that of the intradermal injection (at 120 hrs of injection) (Fig. 6). Also, the tail vein injection with lower dosage has the similar imaging contrast and quality when compared to the intradermal injection with high dosage (Fig. 6A).

ICG agents are known to bind to plasma proteins through the intravenous injection, causing a wavelength shift of up to 25 nm, when compared to ICG in the water (805 nm)^35^. The shift the fluorescence wavelength may be attributed to the microenvironment changes surrounding ICG, which indicates the dynamic association of dyes and tissues. The mechanism behind this phenomenon has yet to be fully understood, as this study is still at the early stage^36^. Underlying mechanisms for the shifts in NIR dental imaging have yet to be understood, which will be a focus of future research.

In summary, our NIR dental imaging system, in combination with the endoscope, can provide more valuable information on dental morphology than that of wide-field imaging. For the optimized imaging conditions, imaging quality can be improved for *in vivo* dental imaging at 72 hrs after ICG injection; intraoral illumination has better imaging contrast than the extraoral illumination; the two injection methods almost have no difference on imaging quality. Due to the small dimension (1–2 mm) of rat molars, the imaging was relatively noisy in the rat dental imaging, but was improved significantly in human tooth (cm in dimension) imaging. ICG-assisted NIR dental imaging can also image human tooth efficiently and obtain the clear profile of the tooth (Fig. 5C). With ICG-aided contrast, the enamel became transparent in NIR I window (800–950 nm), while the dentin was easily distinguished from the enamel (Fig. 5C). The imaging resolution of this method is as good as 100 μm, much better than dental ultrasound^3^. ICG-enhanced NIR dental imaging has the potential to become a safe and real-time *in vivo* imaging tool in dental diagnosis and treatment (surgeries), especially for tooth eruption disorders. In future, NIR dental imaging efficiency could be compared to other imaging techniques, e.g. X-ray. The relevant findings in this study will be extrapolated to large animals and humans for development of an imaging system for human dentistry.

## Methods

### Endoscopic NIR dental imaging system

We designed a miniaturized NIR dental imaging system (spectroscopic device + camera) that is suitable for *in situ* rapid fluorescence imaging in dentistry. The system consists of a laser light source (785 nm laser diode, Turnkey Raman Lasers-785 Series; Ocean Optics, Inc), a spectrometer (QEPro; Ocean Optics, Inc), an imaging module, and a computer (Supplementary Fig. 1).

The laser light source delivers 785 nm to excite ICG, while the spectrometer records the spectrum of ICG fluorescence. The imaging module is composed of a NIR camera (Guppy F038B; Allied Vision Technologies GmbH), in conjunction with an angioscope (Olympus, PF Type 22) for *in vivo* dental imaging, and a sigmoidoscope (Olympus, OSF-3) for *ex vivo* dental imaging. Two filters (bandpass lens: 785 nm, long pass lens: 800 nm; Thorlabs Inc) are used to optimize the detection of ICG spectrum from 800 to 950 nm; a custom-designed bifurcated fiber is to transmit the excitation and emission fluorescence.

### Reagents and animals

ICG powder, bovine serum albumin (BSA, 96%), and phosphate buffered saline (PBS) were purchased from Sigma-Aldrich (St. Louis, MO). Ultrapure water (18.2 MΩ) was used to prepare the reagents throughout this study. For injection, ICG powder was dissolved in ultrapure water with the maximum solubility (1 mg/mL). For the preparation of the standard ICG spectra, the ICG solution was diluted to concentrations ranging from 2 nM to 80 µM (2 nM for each gradient) with 4% BSA-PBS.

Sprague Dawley rats with different postnatal ages were used in this study. A total of eleven P14 (Postnatal 14 days) rats were used for the *in vivo* and *ex vivo* imaging to optimize imaging conditions and study the features of ICG spectrum. Two P9 rats were used to study the effects of the injection methods on the dental imaging, while one P21 rat was used for imaging abnormally shaped molars.

The experimental rats were administered ICG solution by two methods: (1) intradermal injection (from the backside) with 5 mg/kg body-weight; (2) tail vein injection with 10 µL dose per rat (0.3–0.5 mg/kg).

### Ethical statement

All experiments were approved by the Institutional Animal Care and Use Committee of the Louisiana State University (USA) (Protocal#16-117) and in accordance with the ethical guidelines for animal care.

Human tooth samples used for dental imaging were extracted molars collected from the Louisiana State University Health Science Center - Department of Oral & Maxillofacial Surgery (Baton Rouge, USA); the experiment was approved by the Institutional Review Board of Louisiana State University (LSU IRB, IRB#E11061) and in accordance with the ethical guidelines for human samples. This work is an IRB exemption study (category 4a) and the informed consent, waived by the LSU IRB (IRB#E11061), is not required.

### Acquiring rat dental images

The NIR camera, in conjunction with the sigmoidoscope, was used for the *ex vivo* dental imaging, in which the deflecting tip of the sigmoidoscope was fixed at ~4 mm above the rat molar samples. For *in vivo* dental imaging, the deflecting tip of the angioscope with the NIR camera was inserted into the rat’s oral cavity to acquire molar images.

As for the extraoral ICG excitation, the laser fiber head was fixed at about 5 mm below the specimens to excite ICG agents, while the deflecting tip of the endoscope was above the molar of interest. As for the intraoral ICG excitation, the laser fiber was inserted into the working channel of the sigmoidoscope; the deflecting tip imaged the dental structures from the top of the specimens.

To explore spectral properties (such as intensity and peak wavelength) of ICG fluorescence under various NIR dental imaging conditions quantitatively (e.g. imaging windows and injection methods), ICG spectra was tested on the dental tissues in P14 rats by two distinct injection methods. The spectra of dental tissues in P14 rats were recorded from 10 min to 120 hrs after ICG injection.

### Imaging contrast -normalized grayscale difference

To quantitatively analyze the imaging contrast (the difference between two regions of interest), two groups of 10 pixels in the rat dental images were respectively sampled from the molars and surrounding tissues by a designated line. Each group of the pixels was selected from the crests or troughs of the grayscale curve of the designated line. The grayscale of each certain pixel corresponds to the ICG fluorescence intensity recorded at that pixel by the camera. We defined a parameter as *G*_*diff*_, which represents the imaging contrast between the molars and surrounding tissues.

To calculate *G*_*diff*_, the selected pixels were sorted first by equation *sort*(*g*) from the minimum grayscale to the maximum grayscale; then *G*_*diff*_ was calculated by Eq. (2):

1 $$sort(g)=({g}_{min},\,\ldots ,\,{g}_{max})$$

2 $${G}_{diff}=avg(\sum _{i=0}^{9}\,|{\rm{sort}}{({g}_{m})}_{i}-sort{({g}_{s})}_{i}|)/{\rm{avg}}(\sum _{i=0}^{9}\,{g}_{m}+\sum _{i=0}^{9}\,{g}_{s})$$

where *g*_*m*_ and *g*_*s*_ are the grayscales of the pixels from the molars and the surrounding tissues.

## Supplementary information

Supplementary Information SREP-18-15693A

## Data Availability

The datasets generated during and/or analyzed during the current study are available from the corresponding author on reasonable request.
