# Supplementary material for: Indocyanine-green-assisted near-infrared dental imaging - the feasibility of in vivo imaging and the optimization of imaging conditions
Source: Sci Rep. 2019 Jun 3;9:8238. doi: 10.1038/s41598-019-44660-y (PMC6547700; doi:10.1038/s41598-019-44660-y)
Supplement: Supplementary file 1 — Supplementary Information SREP-18-15693A [file 41598_2019_44660_MOESM1_ESM.docx]

**Indocyanine-green-assisted near-infrared dental imaging - the feasibility of *in vivo* imaging and the optimization of imaging conditions**

Zhongqiang Li.^1^, Shaomian Yao^*2^, and Jian Xu^*1^

^1^ Division of Electrical and Computer Engineering, College of Engineering, Louisiana State University, Baton Rouge, LA70803, USA

^2^ Department of Comparative Biomedical Science, School of Veterinary Medicine, Louisiana State University, Baton Rouge, LA70803, USA

**Supplementary Figure 1. The experimental setup of NIR dental imaging.** M1: first molar, M2: second molar.
